# Plastid phylogenomics and green plant phylogeny: almost full circle but not quite there

**DOI:** 10.1186/1741-7007-12-11

**Published:** 2014-02-17

**Authors:** Charles C Davis, Zhenxiang Xi, Sarah Mathews

**Affiliations:** 1 Department of Organismic and Evolutionary Biology, Harvard University, 22 Divinity Ave, Cambridge, MA 02138, USA

## Abstract

A study in *BMC Evolutionary Biology* represents the most comprehensive effort to clarify the phylogeny of green plants using sequences from the plastid genome. This study highlights the strengths and limitations of plastome data for resolving the green plant phylogeny, and points toward an exciting future for plant phylogenetics, during which the vast and largely untapped territory of nuclear genomes will be explored.

## Commentary

The plastid genome, or plastome, has so far been the most important source of data for plant phylogenetics in the era of comparative DNA sequencing. Its utility results from its relatively small size (between 75 and 250 kilobases), largely uniparental inheritance, conservation of gene content and order, and its high copy number in green plant cells. From the early use of a single plastid gene to infer the phylogeny of a broad sampling of seed plants [1], to the now common use of around 80 plastid genes to address finer-scale phylogenetic questions, this circular genome has been a mainstay for evolutionary botanists.

Efforts to understand green plant phylogeny from plastome data have now come full circle. In this issue, Ruhfel *et al*. [2] report results from their analyses of 78 plastid genes from 360 species, from green algae to angiosperms. Their results provide insights into this ongoing effort, adding support for some relationships and highlighting phylogenetic questions that require more data, especially from nuclear genomes.

## Congruence and conflict in plastid phylogenomics

**Figure 1:**
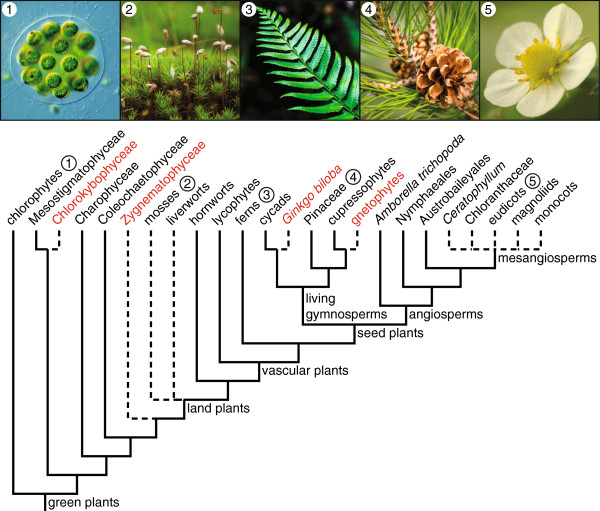
**Simplified green plant phylogeny inferred by Ruhfel *et al*. [**[2]**].** Dashed line branches indicate phylogenetic placements that remain unresolved by the plastome. Relationships that are well resolved in the plastome tree but contentious are highlighted in red. The green algal lineage Klebsormidiophyceae was not included in the plastome study, and thus was not included in the figure.

Ruhfel *et al*. present a phylogeny that is well resolved at most nodes, and largely in agreement with previous studies, including at nodes that have been difficult to resolve (Figure 1). These include the splits between land plants and their algal sister clade [3,4], and between vascular plants and their non-vascular sister clade [5]. Here, Zygnematophyceae, a large clade of mostly freshwater algal species, is identified as sister to land plants. This suggests that shared components of auxin signaling and chloroplast movement likely were present in their common ancestor [3]. Their analyses also support the non-monophyly of bryophytes, or liverworts, mosses and hornworts. These land plants lack a well developed vascular system and have similar ecologies. Hornworts are sister to vascular plants in the plastid tree, consistent with evidence that their sporophytes may be at least partially free-living, unlike those of liverworts and mosses [5].

So how much closer does this new phylogeny bring us to a robust understanding of green plant evolution? This study, like many others, has difficulty resolving key relationships within green plants. This is most evident in the lack of resolution deep in the angiosperm phylogeny among the mesangiosperm clades, *Ceratophyllum*, Chloranthaceae, eudicots, magnoliids, and monocots. Branching order among non-vascular plants, especially involving liverworts and mosses, remains contentious. These problems persist due, in part, to the challenge of placing lineages that are species-poor and divergent in molecular trees, and due to the difficulty of assessing homologies among organisms with very diverse or reduced morphologies.

Despite these few persistent problems, the Rhufel *et al*. tree appears to address many if not all remaining questions. In some cases, however, high support for relationships should be interpreted cautiously because conflicting topologies are supported by other data. Key examples include the previously mentioned sister groups of land plants and vascular plants, but also relationships among major seed plant clades. The latter involves the position of gnetophytes, and the close relationship of cycads and *Ginkgo biloba*. The gnetophytes especially represent a vexing problem in seed plant phylogenetics [6]. They form a small clade of approximately 90 species that are highly divergent from other seed plants in both morphology and molecules. Although plastid phylogenomic studies are converging on the 'Gnecup’ topology, in which gnetophytes are united with cupressophyte conifers, recent nuclear phylogenomic analyses yield the alternative 'Gnepine’ topology in which gnetophytes are united with Pinaceae conifers [7]. Even within individual plastid loci, different nucleotide sites have been shown to favor rival gnetophyte placements [6]. A similarly strong conflict in seed plants concerns the positions of cycads and *Ginkgo biloba*, where their plastid trees strongly unite the two, but other studies place cycads alone as sister to extant gymnosperms.

## Where do we go next?

It is well known that biases within molecular data may be exacerbated in large phylogenomic data sets, leading to erroneous but well supported results, especially when trying to resolve ancient splits. Biases may result from phenomena such as pattern heterogeneity and uneven base frequencies, and their exploration will help us to understand cases of incongruent relationships. For example, when Ruhfel *et al*. accounted for biased GC content, their data placed lycophytes as sister to ferns and seed plants, rather than as the lone sister to seed plants, as in their total evidence tree. Additional approaches for mitigating biases in molecular data sets include increasing taxon sampling and better modeling of nucleotide evolution. Traditional data partitioning schemes try to account for variation in evolutionary rates by partitioning nucleotide sites based on their rate of evolution, most commonly, by gene or codon position. The approach developed by Xi *et al*. [8], in contrast, requires no *a priori* assumptions about evolutionary rates. Instead, the optimal number of partitions, and their contents, are identified using a Bayesian mixture model analysis, which is not influenced by preconceptions about nucleotide evolution. This approach improved resolution over analyses using traditional partitioning strategies and also reduced model complexity because the optimal number of partitions identified in the search was smaller than in commonly used schemes. Ruhfel *et al*. found this scheme to be computationally difficult to implement with their data, but improvements in the efficiency of Bayesian mixture model searches will help. Finally, despite the promise of these improvements to molecular phylogenetic studies, the evolution of green plants cannot be understood from molecular data alone. For example, 70% of seed plant lineages cannot be sampled for molecular datasets because they are extinct. Better integration of morphological evidence from living and fossil taxa are especially needed to reconstruct the evolutionary history of green plants [9].

The largest leap, however, is still ahead of us. Within the green plant species tree there is a 'cloud’ of gene trees [10], of which the plastid genes comprise only a small fraction. An obvious next step is to understand the species tree more thoroughly by incorporating mitochondrial and nuclear data. Mitochondrial data have previously been neglected, but increasingly are being sampled for large-scale phylogenomic studies. However, their informativeness may be limited by slow nucleotide evolution and species relationships may be obscured by potentially rampant horizontal gene transfer involving mitochondrial DNA [11]. Nuclear genomic data, in contrast, have tremendous potential to improve phylogenetic resolution and illuminate the species tree. This source of data will likely reveal surprises when juxtaposed against our current understanding of relationships inferred from plastid data alone. Conflicts between plastid and species trees may result from introgression of the plastid from one species into another, and this may have gone undetected due to heavy reliance on phylogenetic data from uniparentally inherited plastomes. Recombination and gene conversion, which can occur in the plastome, as well as differential selective pressures acting on plastid genes, may also introduce biases and lead to incongruent gene and species trees. Along these lines, recent analyses already indicate that potential plastid-nuclear genome conflicts involve the gnetophytes, early diverging flowering plants, and the large flowering plant orders Lamiales, Malpighiales, and Myrtales. Evaluating the extent to which these incongruent placements demonstrate divergent genome histories requires further exploration, for which the nuclear genome will be a particularly valuable resource.

In addition to providing a wealth of new data for clarifying species trees, the nuclear genome will greatly improve our understanding of important innovations across green plants. Whole genome duplications (WGDs), for example, potentially enhance an organism’s success. Consistent with this, recent analyses of transcriptomes from seed plants indicate that at least three major WGDs occurred very near to the origin of clades characterized by putative key innovations [12,13]. These include the origins of seeds, flowers, and pentamorous floral symmetry - the last of which characterizes more than approximately 70% of all angiosperms (the eudicots) and may be related to their coevolution with bees [14].

Nuclear genomic data also more directly facilitate our ability to connect unique phenotypes with their underlying genetic architectures. In an exemplar study of fungal relationships, Floudas *et al*. [15] investigated the origin of lignin decomposition in fungi - the ability of organisms to degrade lignin synthesized by green plants is a rare feature across the tree of life. This is especially relevant because the absence of lignin decomposition prior to the end of the Carboniferous era (approximately 300 million years ago) accounts for Earth’s extensive stores of fossil fuels. The origin of lignin decomposition by fungi was implicated in the sharp decline in burial of organic carbon around this time. The authors tested this idea by investigating genes implicated in lignin degradation, thus discovering an association between key expansions of these genes coincident with the origins of fungal clades that can degrade lignin. These expansions broadly correspond with the disappearance of fossilized forests from the geological record. Such exemplar studies likely represent the tip of the iceberg, and highlight the tantalizing future research opportunities in plant nuclear genomics.

We are entering into a new and exciting era in plant phylogenetics. Plastid phylogenomics will continue to be a fast and inexpensive way to flesh out the green plant clade, but the next wave is to explore the uncharted terrain of the nuclear genome. It is already on the way, as evidenced by large-scale comparative transcriptome projects (for example [16]) and the growing number of genome sequencing projects focused on phylogenetically key species.
